# Eosinophilic Gastrointestinal Disorders Pathology

**DOI:** 10.3389/fmed.2017.00261

**Published:** 2018-01-15

**Authors:** Margaret H. Collins, Kelley Capocelli, Guang-Yu Yang

**Affiliations:** ^1^Division of Pathology and Laboratory Medicine, Department of Pediatrics, Cincinnati Children’s Hospital Medical Center, Cincinnati, OH, United States; ^2^Department of Pathology and Laboratory Medicine, University of Cincinnati, Cincinnati, OH, United States; ^3^Department of Pathology, Children’s Hospital Colorado, Aurora, CO, United States; ^4^Department of Pathology, University of Colorado, Denver, CO, United States; ^5^Department of Pathology, Feinberg School of Medicine, Northwestern University, Chicago, IL, United States

**Keywords:** esophagitis, colitis, inflammatory bowel disease, allergy, genome

## Abstract

Eosinophilic gastrointestinal disorders (EGID) are characterized pathologically by excess eosinophils in mucosal biopsies of one or multiple sites in the gastrointestinal (GI) tract, simultaneously or sequentially. Eosinophilic esophagitis (EoE) is the best characterized EGID, and in most patients it is an abnormal immune-mediated response to food antigens. Current recommendations for diagnosis include signs and symptoms of esophageal dysfunction that do not respond to proton-pump inhibitor therapy, and esophageal biopsies that exhibit at least 15 intraepithelial eosinophils in at least one high power field (HPF). Therapy consists of swallowed glucocorticoids or dietary elimination. Eosinophilic gastritis (EG) is the second most common form of EGID, but like all forms of EGID except EoE consensus recommendations for either clinical or pathological diagnosis do not exist. EG may be associated clinically with peripheral blood eosinophilia, hypoalbuminemia, and anemia, and pathologically with marked expansion of lamina propria by dense eosinophilic infiltrates. Eosinophilic enteritis (EE) may be subdivided into eosinophilic duodenitis, eosinophilic jejunitis, and eosinophilic ileitis. Most investigators believe that EE rarely, if ever, exists as a solitary form of EGID and is encountered only in patients who have at least one other affected portion of the GI tract. Eosinophilic colitis (EC) is perhaps the most enigmatic EGID. Distinction of EC from inflammatory bowel disease may be problematic especially in children. Multiple possible etiologies for EGID include hypereosinophilic syndrome, drug reactions, etc. Currently, the only etiology that can be identified histologically is parasitic infestation, if a portion of an invasive parasite is found in mucosal biopsies. This review will provide guidelines for the pathologic diagnosis of the various forms of EGID.

## Introduction

In the mid-twentieth century, excess eosinophils in the gastrointestinal (GI) tract were correlated with a multitude of symptoms, based on the examination of resected bowel segments. Increased density of eosinophils in mucosa was found in resected bowels from patients who manifested anemia, hypoproteinemia, and diarrhea, and increased density of eosinophils in the muscularis propria was seen in resected specimens from patients whose major clinical manifestation was bowel obstruction (1). The development of safe and flexible endoscopes resulted in fewer surgical procedures, and therefore resected bowel segments, and greater reliance on mucosal biopsies for diagnosis of and monitoring response to therapy for GI diseases. Currently, eosinophilic gastrointestinal disorders (EGID) are defined pathologically, virtually, and exclusively by endoscopically obtained mucosal biopsies (2, 3) necessitating greater understanding of the role of eosinophils in GI mucosal health and disease (4, 5). Pathologic confirmation of eosinophilic inflammation confined to the muscularis propria can be accomplished currently by laparoscopic mural bowel biopsies that are guided by imaging studies showing bowel wall thickening. There may also be subserosal dense eosinophil infiltrates that are associated with ascites (1, 6), and in those cases large numbers of eosinophils are found in the ascitic fluid.

Eosinophilic gastrointestinal disorders are subclassified according to the affected site(s) as eosinophilic esophagitis (EoE), eosinophilic gastritis (EG), eosinophilic enteritis (EE), and eosinophilic colitis (EC). Eosinophils are normally found in the mucosa of all parts of the GI tract except the esophagus, but there are few studies that quantify eosinophils in normal GI mucosa complicating the ability to recognize pathologic numbers of eosinophils (7). Consensus recommendations for diagnosis of EGID currently exist only for EoE.

## Eosinophilic Esophagitis

In the late-twentieth century, pediatric and adult patients who had abundant eosinophils in esophageal mucosal biopsies and who responded clinically and histologically to dietary restrictions were described (8–10). Characteristic esophageal endoscopic findings were reported in such patients (11). Case reports and small series of affected patients appeared increasingly in the literature. Subsequent retrospective studies of archived pathology slides identified esophageal biopsies, displaying numerous intraepithelial eosinophils in files from the 1980s (12–14). Some of those patients were later diagnosed with EoE, and patients who had as few as 5 eosinophils/HPF were statistically more likely to have signs and symptoms of esophageal dysfunction years later compared to patients whose esophageal biopsies had not displayed intraepithelial eosinophils in the remote past (15).

**Figure 1:**
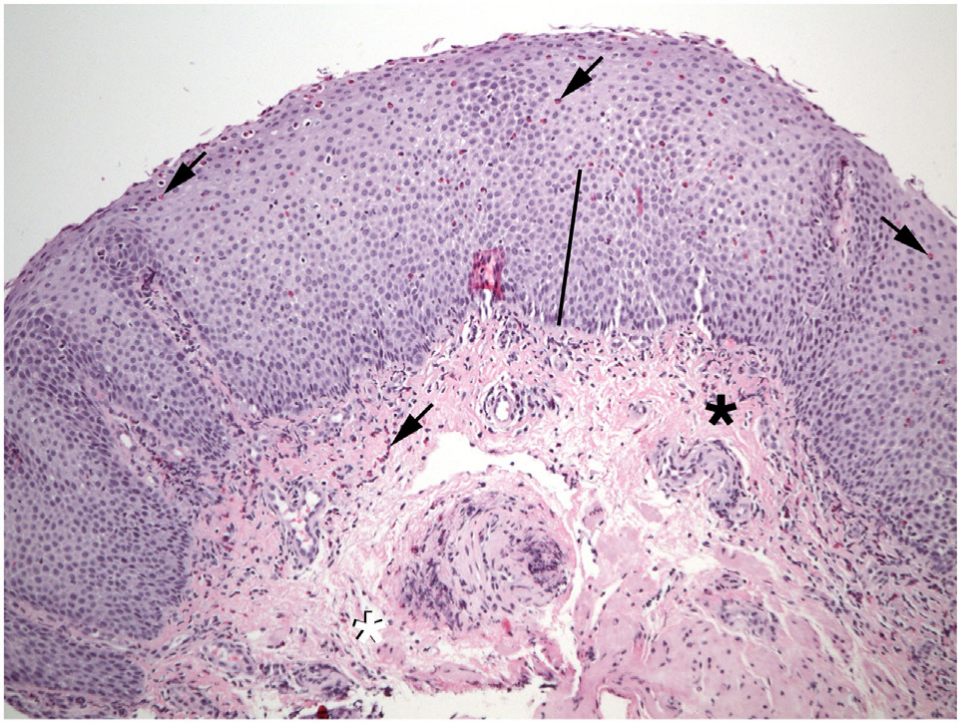
Numerous eosinophils (arrows) are found in the epithelium of this esophageal biopsy. The basal zone is markedly expanded (bar). Lamina propria fibers appear thickened near the epithelium (black asterisk), but not at the deep margin (white asterisk). Eosinophils are also present in the lamina propria (shaded arrows).

**Figure 2:**
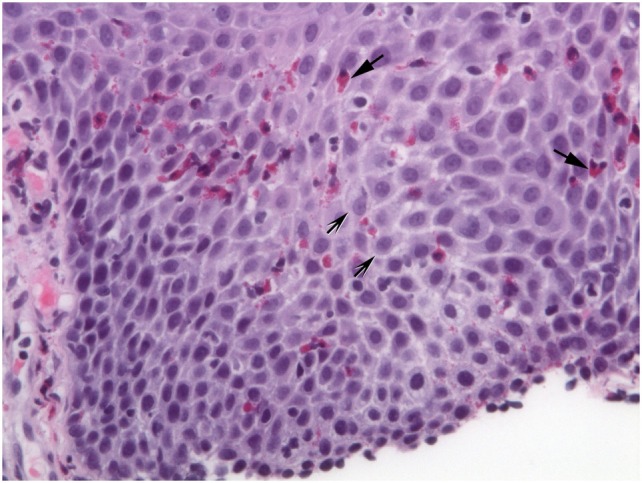
Extracellular eosinophil granules are seen (arrows). Intercellular bridges (shaded arrows) are visible in the dilated intercellular spaces.

In 2007, the first set of recommendations for EoE diagnosis and therapy was published (16), and several have subsequently appeared (17–19). All guidelines or recommendations cite a peak eosinophil count of ≥15 eosinophils in at least one high power field (HPF) in an esophageal biopsy from at least one site in the esophagus (distal, mid, or proximal) as the pathologic criterion for diagnosis. All recognize that other pathologic changes are found in EoE biopsies. Recently, an EoE histology scoring system (EoEHSS) was developed that scores eight pathologic features including eosinophil density, but also pathologic features whose definition does not include eosinophils (20). Eosinophil inflammation, basal zone hyperplasia, eosinophil abscess, eosinophil surface layering, dilated intercellular spaces, surface epithelial alteration, dyskeratotic epithelial cells, and lamina propria fibrosis are evaluated in the EoEHSS (Figures 1 and 2; Table 1). The features are scored separately for severity of change (grade) and for the amount of tissue that is affected by each feature (stage). The EoEHSS scores better identified treated compared to untreated subjects’ biopsies than did peak eosinophil count, indicating the importance of evaluating more than eosinophil density in esophageal biopsies obtained to diagnose or monitor EoE, and also indicating the importance of determining the amount of tissue damage in addition to the degree of damage. A systematic method to evaluate the myriad endoscopic features of EoE has also been developed (21).

**Table 1:** Eosinophilic esophagitis (EoE) histology scoring system definitions.

Feature	Definition
Eosinophilic inflammation	Based on peak eosinophil count
Basal zone hyperplasia	Basal zone occupies more than 15% of total epithelial thickness
Eosinophil abscess	Eosinophil aggregate that disrupts the underlying epithelial architecture
Eosinophil surface layering	Eosinophils align in one or more rows in the upper third of the epithelium
Dilated intercellular spaces	Intercellular bridges are visible in paracellular spaces
Surface epithelial alteration	Surface epithelial cells stain more darkly than normal and eosinophils that may be present among the altered epithelial cells
Dyskeratotic epithelial cells	Epithelial cells with deeply staining cytoplasm and shrunken hyperchromatic nuclei that generally occur singly and may be found anywhere in the epithelium
Lamina propria fibrosis	Coalesced fibrils form fibers of varying diameter

A whole-genome messenger RNA esophageal expression analysis identified a unique EoE transcriptome and eotaxin-3 was the most upregulated gene (22). A diagnostic panel derived from the results of the original microarray study and consisting of 96 genes distinguishes EoE from non-EoE biopsies and can be used on paraffin-embedded tissue samples (23).

Prior to treatment, children who have EoE commonly experience vomiting and poor weight gain or weight loss, but adolescents and adults commonly experience dysphagia that may include food impaction (24). Therapy commonly consists of swallowed glucocorticoids (25–30) and elimination diet (31, 32). Response to dietary antigen-removal suggests that Th2 immunity is important in EoE pathogenesis; in fact, IL-13 and IL-5 levels are increased in EoE biopsies (33), and monoclonal antibodies to each of those cytokines reduce esophageal inflammation (34–38). Proton pump inhibitors (PPI) were used to distinguish EoE patients from those who had gastroesophageal reflux disease (GERD): clinical response to a PPI was considered not consistent with EoE and patients were believed to have GERD (16). However, a group of patients who respond initially to PPI, but subsequently may become refractory to PPI emerged and is believed to be a phenotype of EoE (39). Their pre-PPI biopsies are indistinguishable from patients who do not respond to PPI therapy and the genotype identified in their biopsies is very similar to nonresponders (40).

Eosinophilic esophagitis has increased in both prevalence and incidence (41–43) and is currently an important cause for food bolus impaction (44) and esophagitis (41, 45). Care of afflicted patients in the U.S. is estimated to consume approximately one billion dollars annually (46).

## Eosinophilic Gastritis

**Figure 3:**
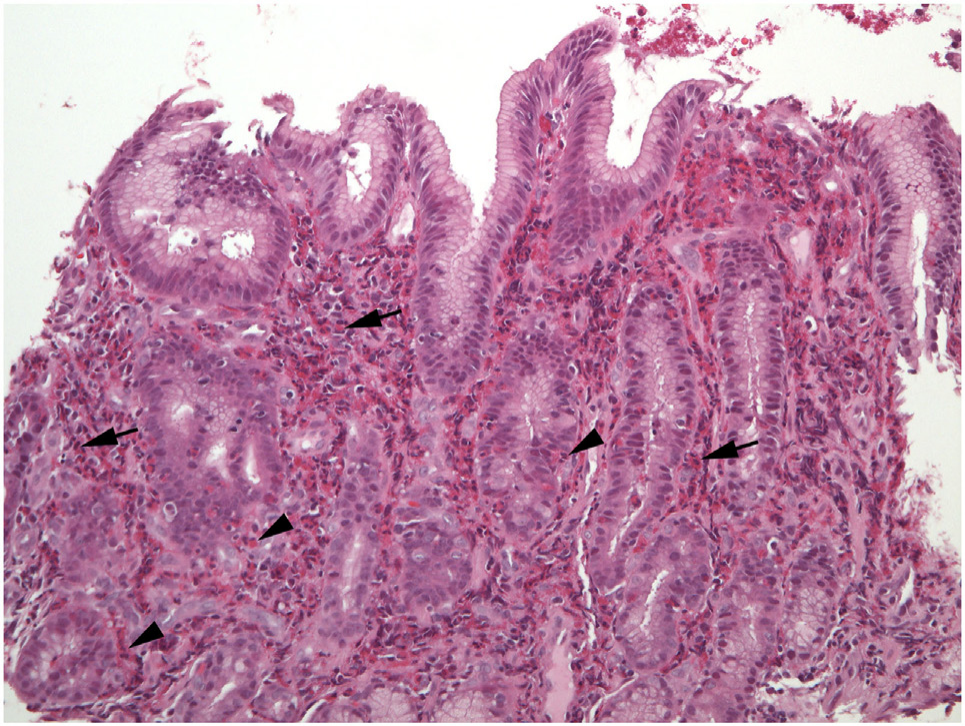
The lamina propria of this section of gastric mucosa is almost entirely occupied by sheets of eosinophils (arrows). Numerous intraepithelial eosinophils are found in gland epithelium (arrowheads).

In contrast to the esophagus, eosinophils are normally present in gastric mucosa, but in lower concentrations than in the small and large bowel (47–49). The criteria for diagnosing EG histologically include ≥30/HPF in ≥5 HPF and ≥70/HPF in ≥3 HPF (49, 50). Common features of EG biopsies are eosinophil sheets in expanded lamina propria, excess intraepithelial eosinophils, eosinophil cryptitis/abscess, and eosinophils in the muscularis mucosa and submucosa (Figure 3). Mast cells and FOXP3-positive lymphocytes are more abundant in EG biopsies compared to controls (51).

An EG transcriptome overlaps minimally with the EoE transcriptome (52). However, cadherin 26 (CDH26) is the most upregulated gene in EG and is also among the most upregulated genes in EoE. CDH26 is expressed by esophageal and gastric epithelial cells in EoE and EG respectively, binds to α4 and αE integrins, and regulates leukocyte adhesion and activation. Importantly, CDH26 inhibits CD4+ T-cells *in vitro*, suggesting a role as a downregulator of inflammation in EGID (52).

Clinical features of EG include epigastric pain, peripheral blood eosinophilia, anemia, and hypoalbuminemia (49–51). Endoscopic abnormalities include nodular mucosa, erythema, and ulcers/erosions, but the mucosa may appear normal (49–51). EG may occur in isolation, or may be associated with excess eosinophil infiltrates in the rest of the GI tract, especially the esophagus (50, 51), either simultaneously or sequentially. Antigen restriction successfully reduces symptoms and tissue eosinophilia in some pediatric EG patients (50).

## Eosinophilic Enteritis

**Figure 4:**
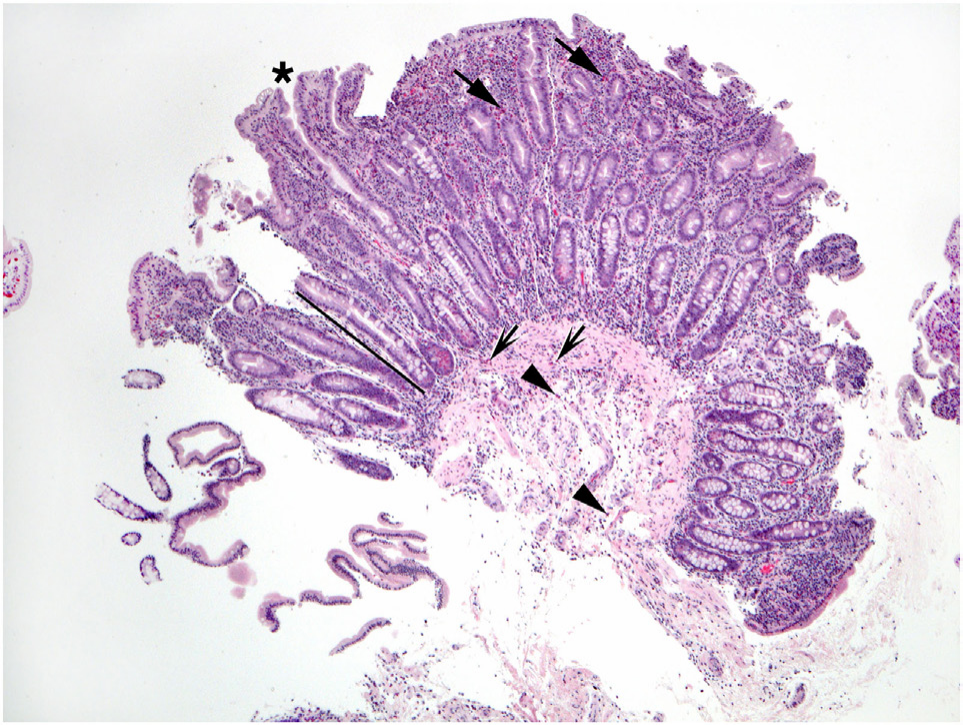
This duodenal biopsy shows few preserved short villi (asterisk), elongated crypts (bar), and numerous eosinophils in the lamina propria (arrows), muscularis mucosa (shaded arrows), and submucosa (arrowheads).

**Figure 5:**
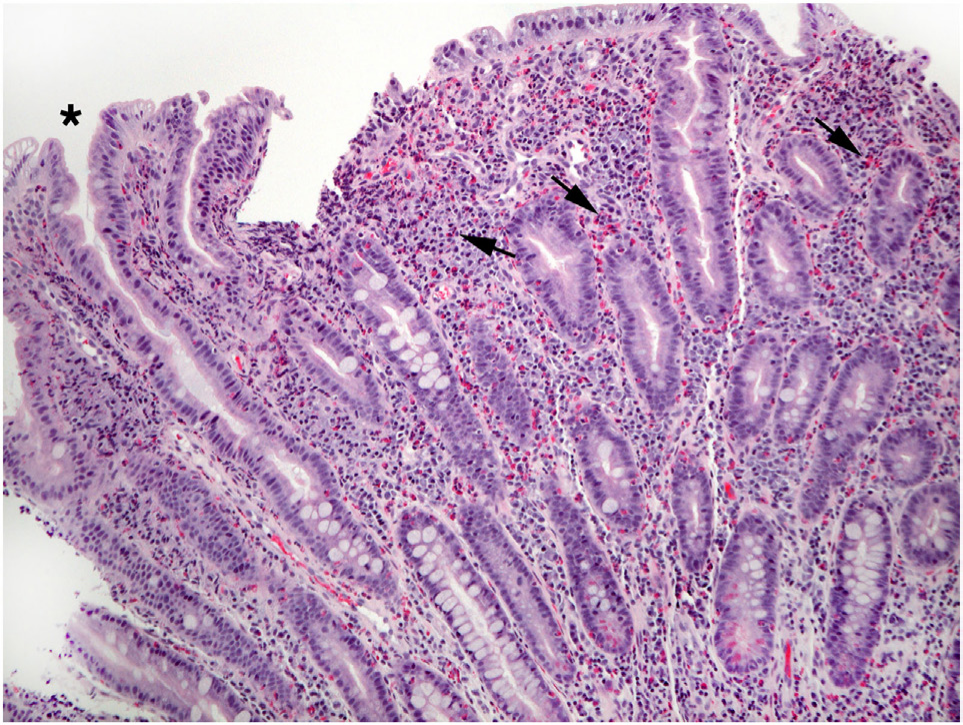
This view of Figure 4 illustrates blunt villi (asterisk) and confirms numerous lamina propria eosinophils (arrows).

**Figure 6:**
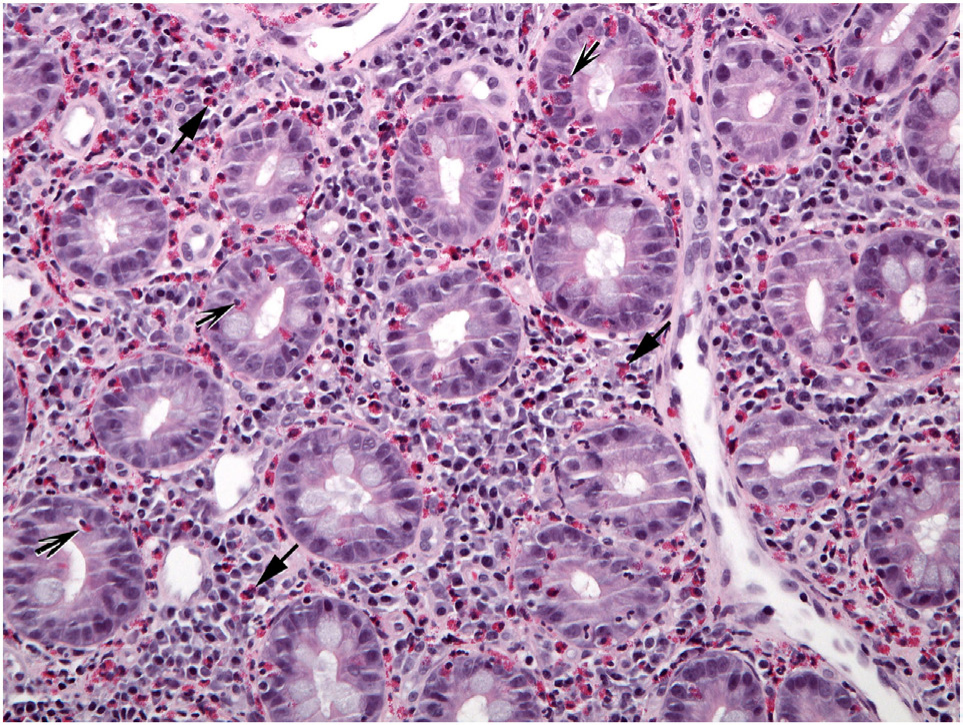
A different biopsy shows numerous eosinophils in duodenal lamina propria (arrows) and crypt epithelium (shaded arrows).

Excess eosinophils in the small intestine could be considered a multiple of the maximum count found in normal biopsies, such as 2 × 26/HPF or 52/HPF in duodenal mucosa, and 2 × 28/HPF or 56/HPF in ileum (7). Excess eosinophils restricted to the small intestine appear to be exceedingly rare, i.e., small bowel mucosal eosinophilia appears to be commonly, perhaps exclusively, associated with excess eosinophil density in the mucosa of other parts of the GI tract. Patients who report dyspepsia and do not have ulcers have increased numbers of duodenal mucosal eosinophils compared to patients who do not report dyspepsia (53). Increased numbers of eosinophils in the lamina propria, increased intraepithelial eosinophils compared to normal counts (48), blunt villi (54), and eosinophils in the muscularis mucosa and submucosa may be found in EE (Figures 4–6). Parts of invasive helminths may be found in small bowel mucosal biopsies permitting identification of a specific cause for excess mucosal eosinophils (55).

## Eosinophilic Colitis

**Figure 7:**
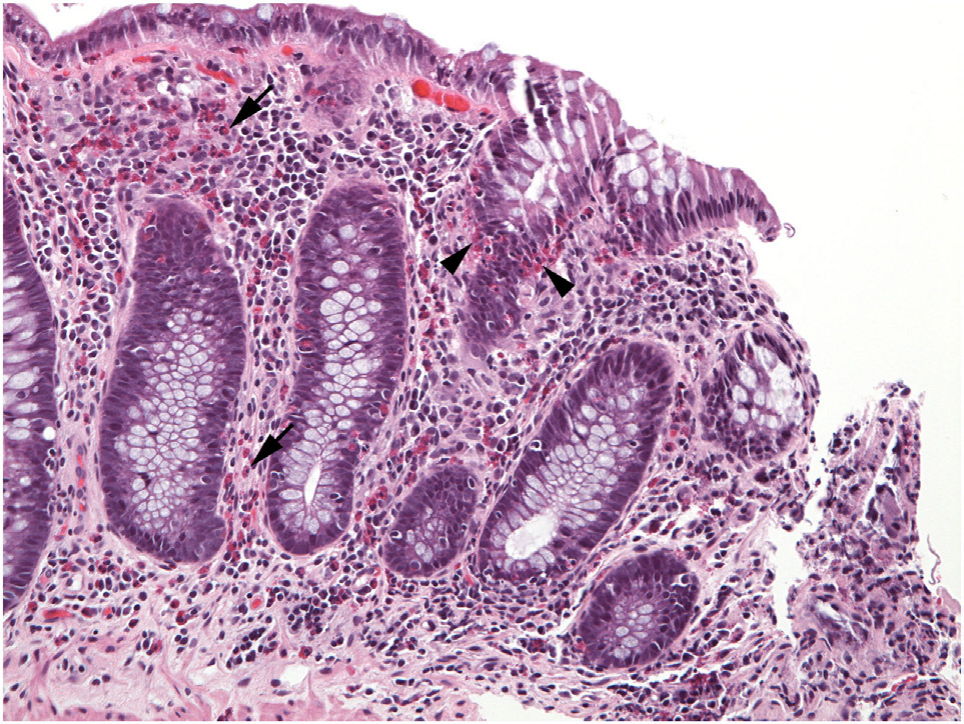
Numerous eosinophils populate the lamina propria (arrows) in this well-oriented section of colonic mucosa and also invade crypt epithelium (arrowheads).

**Figure 8:**
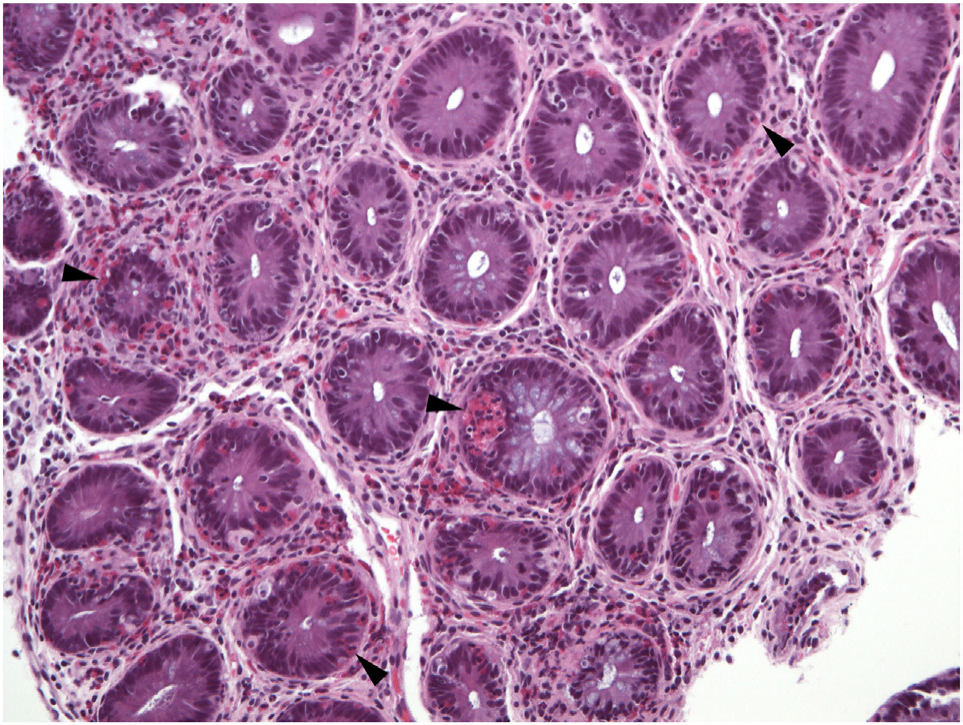
Virtually all crypts in this field of a colon biopsy display increased numbers of intraepithelial eosinophils (arrowheads).

Eosinophils are normally present in colon mucosa and are most abundant in the right colon of both children (47, 48, 56) and adults (57). Therefore, using a single threshold value to identify increased eosinophil density is less accurate and potentially misleading compared to applying threshold values appropriate for each colon site (right, transverse, left, rectosigmoid) to properly labeled and separately submitted colon mucosal biopsies. Excess eosinophils could be considered a multiple of the peak count/HPF in normal biopsies, including 2 × 50/HPF or 100/HPF in cecum and ascending colon, 2 × 42/HPF or 84/HPF in transverse and descending colon, and 2 × 32/HPF or 64/HPF in rectosigmoid mucosa (7). Histologic features in colon biopsies showing increased eosinophil density include eosinophil cryptitis/crypt abscesses, crypt architectural abnormalities, increased intraepithelial eosinophils, and eosinophils in muscularis mucosa and submucosa (Figures 7 and 8) (57–63).

The prevalence of EC is difficult to ascertain, partly, because guidelines for clinical or pathologic diagnosis do not exist. Nevertheless, a reasonable approach is that EC should be a clinicopathologic diagnosis, akin to EoE, requiring both symptoms referable to colonic dysfunction and colon biopsies showing excess eosinophils. Colon biopsies that displayed eosinophil density greater than normal for site plus six SDs from 194 patients were identified in a pathology database of 1.2 million patients (prevalence 1:6,000) (57). Most of those patients had symptoms, mainly diarrhea and abdominal pain, but approximately one-third were asymptomatic. Reported endoscopic abnormalities included erythema, erosions, whitish elevated lesions, pale granular mucosa, and aphthous ulcers.

Several studies have documented that excess eosinophils may be found in mucosal biopsies from patients who have IBD (57, 61, 64, 65). Indeed eosinophils may be more numerous in biopsies from children who have IBD compared to biopsies from children with allergic conditions (66), and elevated eotaxin-1 levels are reported in rectosigmoid biopsies from children with ulcerative colitis (64). The presence of acute inflammatory cells in colon biopsies showing chronic changes and excess eosinophils, especially those lacking sheets of eosinophils, should raise suspicion for IBD with excess eosinophils as the correct diagnosis. More abundant co-localization of IgE deposits with tryptase deposits in perineural locations may distinguish EC biopsies from biopsies of patients who have IBD with excess eosinophils (67).

The role of eosinophils in IBD is not clear. Recently, however, increased mucosal expression of genes that mediate type 2 and type 17 immune responses were shown to distinguish UC at baseline from colon-only Crohn disease at diagnosis, and high IL-13 expression was found in patients who subsequently exhibited improved clinical outcome (68).

Increased numbers of eosinophils may be found in colon biopsies of immunosuppressed patients, especially those receiving tacrolimus, who have had an organ transplant (69–71) which may resolve with food restriction.

Few studies quantify mast cells in the colon (56, 72), but colonic biopsies that show apparently increased numbers of mast cells, either as part of mastocytic enterocolitis (73, 74) or as part of systemic mastocytosis (75, 76), also show increased numbers of eosinophils.

Allergic colitis of infancy has been diagnosed if >20 eosinophils/HPF are found in rectal biopsies (77) which may be in a patchy distribution (78), which resolves clinically following removal of the offending antigens, typically cow’s milk, from an affected infant’s diet. Since this condition is so easily, and apparently permanently, treated by withdrawal of a single food substance from the diet, classification as an EGID is considered inappropriate by some experts.

The differential diagnosis for increased eosinophil density in colon mucosa is more extensive than discussed above, and includes parasitic infections, hypereosinophilic syndrome, etc. Primary EC is a diagnosis made only after all known causes for increased mucosal colon eosinophils have been eliminated (58) (Table 2).

**Table 2:** Underlying diseases associated with gastrointestinal (GI) mucosal eosinophilia.

Underlying disease	Affected GI site
Food allergy	Any site
Gastroesophageal reflux disease	Esophagus
Inflammatory bowel disease	Small intestine, colon
Parasitic infections	Any site, e.g., stomach, small intestine (Anasakis, *Helicobacter pylori*), proximal small intestine (*Strongyloides stercoralis, Giardia*), small intestine, colon (*Cryptosporidium)*, small intestine (*Ascaris lumbricoides*), colon (*Entamoeba histolytica, Dientamoeba fragilis, Blastocystis* species, *Balantidium coli, Trichuris trichiura*), distal small intestine (*Angiostrongylus costaricensis*), proximal colon, and appendix (*Enterobius vermicularis*)
Drug reactions	Any site including medication-induced (“pill-induced”) esophagitis
Systemic mastocytosis	Small and large intestine
Neoplasm, e.g., leiomyomatosis, granular cell tumor	Esophagus
Vasculitis e. g., eosinophilic granulomatosis with vasculitis (Churg-Strauss syndrome), granulomatosis with polyangiitis, microscopic polyangiitis	Any site
Connective tissue disease (e. g., systemic sclerosis)	Any site
Hypereosinophilic syndrome	Any site
Celiac disease	Esophagus, duodenum
Organ transplant	Any site

## Eosinophilic Gastroenteritis (EGE)

This term is used in multiple ways and unfortunately may indicate excess eosinophils in one or more than one part of the GI tract. Use of site-specific terminology, such as EG for eosinophilia restricted to the stomach, would help to bring greater clarity than using a term that is less specific.

In children with allergic EGE defined as excess eosinophils in either gastric or duodenal mucosa, significantly greater numbers of mast cells were found in duodenal but not gastric biopsies from patients who had associated protein-losing enteropathy compared to those who did not (79). All patients responded well to amino-acid-based formula, but food hypersensitivities did not completely resolve.

## Future Directions

Many aspects of EGID diagnosis, pathogenesis, therapy, etc., remain to be determined. Recently, the NIH funded the Consortium of Eosinophilic Gastrointestinal Researchers (CEGIR) to facilitate studies of these rare diseases. CEGIR conducts observational and interventional studies of EGID subjects that include correlative studies of clinical, pathological, molecular, genetic, and microbiomic components of these diseases. These studies will hopefully yield basic science and clinical knowledge that will lead to novel and effective therapies.

## Author Contributions

MC, KC, and G-YY: substantial contribution to conception and design of work; drafting and revising for intellectual content; final approval of version to be published; and agreement to be accountable for all aspects of the work.

## Conflict of Interest Statement

MC has received funding for central pathology review in clinical trials to treat eosinophilic esophagitis from Meritage, Shire, Receptos, and Regeneron. The remaining coauthors declare that the research was conducted in the absence of any commercial or financial relationships that could be construed as a potential conflict of interest.
